# Short-Term Effect of Antibiotics on Human Gut Microbiota

**DOI:** 10.1371/journal.pone.0095476

**Published:** 2014-04-18

**Authors:** Suchita Panda, Ismail El khader, Francesc Casellas, Josefa López Vivancos, Montserrat García Cors, Alba Santiago, Silvia Cuenca, Francisco Guarner, Chaysavanh Manichanh

**Affiliations:** 1 Digestive System Research Unit, Vall d'Hebron Research Institute, Barcelona, Spain; 2 Centro de Investigación Biomédica en Red en el Área temática de Enfermedades Hepáticas y Digestivas (CIBERehd), Instituto de Salud Carlos III, Madrid, Spain; 3 Internal Medicine Department, Capio Hospital General de Catalunya, Universitat Internacional de Catalunya, Barcelona, Spain; Teagasc Food Research Centre, Ireland

## Abstract

From birth onwards, the human gut microbiota rapidly increases in diversity and reaches an adult-like stage at three years of age. After this age, the composition may fluctuate in response to external factors such as antibiotics. Previous studies have shown that resilience is not complete months after cessation of the antibiotic intake. However, little is known about the short-term effects of antibiotic intake on the gut microbial community. Here we examined the load and composition of the fecal microbiota immediately after treatment in 21 patients, who received broad-spectrum antibiotics such as fluoroquinolones and β-lactams. A fecal sample was collected from all participants before treatment and one week after for microbial load and community composition analyses by quantitative PCR and pyrosequencing of the 16S rRNA gene, respectively. Fluoroquinolones and β-lactams significantly decreased microbial diversity by 25% and reduced the core phylogenetic microbiota from 29 to 12 taxa. However, at the phylum level, these antibiotics increased the Bacteroidetes/Firmicutes ratio (*p* = 0.0007, *FDR* = 0.002). At the species level, our findings unexpectedly revealed that both antibiotic types increased the proportion of several unknown taxa belonging to the Bacteroides genus, a Gram-negative group of bacteria (*p* = 0.0003, *FDR*<0.016). Furthermore, the average microbial load was affected by the treatment. Indeed, the β-lactams increased it significantly by two-fold (*p* = 0.04). The maintenance of or possible increase detected in microbial load and the selection of Gram-negative over Gram-positive bacteria breaks the idea generally held about the effect of broad-spectrum antibiotics on gut microbiota.

## Introduction

Clinicians commonly prescribe antibiotics to treat infections. The choice of antibiotic is well indicated in clinical guidelines for targeting specific pathogens, Gram-positive or Gram-negative bacteria [1]. However, little is known about the effects of antibiotics on the whole composition and load of the gut microbiota immediately after treatment.

Human fecal microbiota is composed of four main groups of bacteria (phyla), namely Firmicutes, Bacteroidetes, Proteobacteria and Actinobacteria [2], the first two phyla accounting for more than 80% of the microbiota. Firmicutes comprise mostly Gram-positive bacteria with a DNA that has a low G+C content, but also include Gram-negative bacteria. Bacteroidetes include Gram-negative bacteria, which are represented mainly by the *Bacteroides* genus in the human gut. Proteobacteria consist of Gram-negative bacteria and include a wide variety of well-studied pathogens. Actinobacteria are a group of Gram-positive bacteria with a DNA that has a high G+C content.

Since the introduction of antibiotics in the 1940s, the short-term effect of these drugs on gut microbiota has been mainly documented on the basis of culture methods. However, given the difficulty in developing cultures for most gut bacteria [3], the information gathered from this technique is insufficient to understand the full targets of antibiotics. A few recent studies have used high-throughput sequencing technology to deeply characterize the long-term effect of antibiotics [4], [5], [6], [7]. These studies have shown that treatment is followed by a significant alteration of the gut microbiota composition and a decrease between one-fourth to one-third of the microbial diversity in the digestive tract [4], [8]. The microbiota is relatively resilient and returns to the pre-treatment state several weeks after drug cessation [9].

However, other recent studies on the long-term effects of antibiotic intake have shown that microbiota does not show complete resilience three months after treatment cessation [4], [5], [9], [10], [11]. Variations in the resilience observed might be due to differences in the methodology used to analyze microbiota variability: TGGE [9] versus high-throughput sequencing technique [4], [5], [10] (**Table S1**).

In experimental models, up to a 10-fold reduction in bacterial 16S rDNA was detected (qPCR) immediately after treatment with antibiotics [10], [12]. Furthermore, Hill et al. showed that bacterial depletion was associated with anatomic, histologic, and immunologic changes characteristic of reduced microbial stimulation [12]. Indeed, for latter associated effect, the authors showed that transcript levels of ifng and il17a genes, coding for IFNγ or IL-17A, were significantly reduced in the small intestine of antibiotic-treated animals as compared to controls, thus demonstrating that microbial signals participate in the maintenance of normal intestinal effector T lymphocyte populations. However, as far as we know, no data in human adults has yet been reported regarding microbial load combined with microbial composition analysis before and immediately after antibiotic intake.

Using quantitative real-time PCR (qPCR) and high-throughput sequencing techniques, here we describe the short-term effect of antibiotics on the composition, structure and load of the gut microbial community of patients who received a seven-day treatment with commonly used antibiotics.

## Materials and Methods

### Ethics Statement

This study was approved by the Institutional Review Board of the Capio Hospital General de Catalunya, Barcelona, Spain. Participants provided their written consent to participate in this study.

### Patients and sample collection

Twenty-one participants (from 18 to 80 years old), who were admitted to the hospital for non-digestive diseases (bronchitis, urinary tract diseases, pneumonia, bacteraemia or prostatitis), were recruited to donate stool samples before and after seven-day course of antibiotics. Patients were treated with commonly used antibiotics (β-lactams (N = 11) and fluoroquinolones (N = 10)): amoxicillin-clavulanate (amoxiclav) (N = 7), levofloxacin alone (N = 8) or in combination with metronidazole (N = 1), ceftriaxone alone (N = 1) or in combination with azithromycin (N = 2), ciprofloxacin (N = 1), and piperacilin/tazobactam (N = 1). The dose of antibiotic was adjusted to the etiology of the infection and patient characteristics, following current clinical guidelines. Participants had not received antibiotics during 2 months prior to the study. For microbial composition analyses, stool samples were collected before and on the seventh day of the antibiotic treatment and were stored immediately at −20°C in a home freezer and transported afterwards in a freezer pack to the laboratory.

### Microbial community analyses

#### Genomic DNA extraction

A frozen aliquot (200 mg) of each fecal sample was suspended in 250 µl of guanidine thiocyanate, 0.1 M Tris (pH 7.5) and 40 µl of 10% N-lauryl sarcosine. Genomic DNA was extracted as described by Godon et al. [13].

Pyrosequencing of the V4 variable region of the 16S rRNA gene. Extracted DNA was subjected to PCR-amplification of the V4 region of the bacterial and archaeal 16S rRNA gene (16S ribosomal RNA). On the basis of our analysis done using PrimerProspector software [14], the V4 primer pairs used in this study were expected to amplify almost 100% of the Archaea and Bacteria domains. The 5′ ends of the forward (V4F_517_17: 5′- GCCAGCAGCCGCGGTAA -3′) and reverse (V4R_805_19: 5′- GACTACCAGGGTATCTAAT -3′) primers targeting the 16S gene were tagged with specific sequences for pyrosequencing as follows: 5′-CCATCTCATCCCTGCGTGTCTCCGACTCAG-{MID}-{GCCAGCAGCCGCGGTAA}-3′ and 5′- CCTATCCCCTGTGTGCCTTGGCAGTCTCAG-{GACTACCAGGGTATCTAAT}-3′. Tag pyrosequencing was performed using multiplex identifiers (MIDs) of 10 bases provided by Roche, which were specified upstream of the forward primer sequence (V4F_517_17). Standard PCR (1 unit of *Taq* polymerase (Roche) and 20 pmol/µL of the forward and reverse primers), was run in a Mastercycler gradient (Eppendorf) at 94°C for 2 min, followed by 35 cycles of 94°C for 30 sec, 56°C for 20 sec, 72°C for 40 sec, and a final cycle of 72°C for 7 min [15]. The 16S rRNA V4 amplicons were subsequently sequenced on a 454 Life Sciences (Roche) Junior system (Scientific and Technical Support Unit, Vall d'Hebron Research Institute, Barcelona, Spain), following standard 454 platform protocols.

#### Sequence analysis

The sequences were analyzed using the QIIME pipeline [16] and have been deposited in the NIH Short Read Archive under accession number SRP035398. From the pyrosequencing, 159,536 high quality sequences of 290 base pairs (bp) on average were recovered from 42 samples (with an average of 3945 sequences per sample), after filtering high quality reads, as previously described [10]. From these 42 samples, we obtained 2171 taxa (or molecular species). After removing taxa with low abundance (i.e. in order to avoid false positive OTUs, we considered only taxa that representing at least 0.2% of the microbial community in at least one of the 42 samples), we recovered 427 microbial taxa.

Rarefaction analysis was done for all samples, with 10 repetitions using a step size of 100, from 100 to 2000 sequences per sample. For β diversity analyses, which examine changes between microbial communities, sequence data were normalized at 2091 sequences per sample, excluding one sample that contained only 1331 sequences. The sequencing depth was evaluated by generating a rarefaction curve based on the number of estimated species (Chao1 estimator of species richness) [17] and the number of sequences per sample, and by applying Good's coverage formula [18]: 1-(n/N)]*100, where n is the number of taxa in a sample represented by a singleton and N is the total number of sequences in the sample (N = 2091 sequence per sample). These estimators allow researchers to gain insight into how the limited sampling relates to the entire community sampled. The rarefaction curves (**Figure S1**), which were calculated for each sequence data set (before and after antibiotic treatment), showed that richness almost reached a plateau with 2000 sequence reads. Good's coverage, calculated for each sample of this study, allowed us to recover an average value of 98.26%, indicating that any new sequence generated had only a 1.74% chance of corresponding to a new taxon. Caporaso et al [19] demonstrated that this depth of sequencing is sufficient to capture the same relationship among samples as with 3.1 million reads per sample. The principal coordinates analysis (PCoA) was performed on pairwise unweighted and weighted UniFrac distances [20].

#### Microbial load assessment

In order to assess the microbial load, the extracted DNA was used to amplify the V4 region of the 16S rRNA gene by quantitative real-time PCR (qPCR) using the above-cited primers (V4F_517_17 and V4R_805_19). The qPCR was performed with the 7500 Fast Real-Time PCR System (Applied Biosystems) using optical-grade 96-well plates. The PCR reaction was performed in a total volume of 25 µl using the Power SYBR Green PCR Master Mix (Applied Biosystems), containing 100 nM of each of the universal forward and reverse primers. The reaction conditions for amplification of DNA were 50°C for 2 min, 95°C for 10 min, and 40 cycles of 95°C for 15 sec and 60°C for 1 min. All reactions were performed in triplicate and mean values were calculated. This experiment was also duplicated to ensure accuracy. Mean values of both experiments were taken into account. Data were analyzed using Sequence Detection Software version 1.4, supplied by Applied Biosystems.

#### Statistical analysis

The D'Agostino-Pearson omnibus normality test was used to check the normality of data distribution. Comparisons of parametric normally distributed data were made by the paired t-test for intra-group comparisons; otherwise the Wilcoxon matched-pairs signed-rank test was used. *p* values <0.05 were considered significant.

We used the otu_category_significance.py script from the QIIME pipeline and ANOVA to test which taxa were associated with the use of the antibiotics. This analysis provided the *FDR* value, which is defined to be the false discovery rate of the *p* value (corrected *p* value) and is considered significant when <0.1 [21].

## Results

### Microbiota composition before treatment

Twenty-one patients (18 men, 3 women; median age: 69 years), who were admitted to the hospital for bronchial infection (N = 15), urinary infection (N = 1) or other infections (pneumonia, bacteraemia or prostatitis; N = 5), were enrolled in the study. They took antibiotics (or antibiotic combinations) for seven days and provided fecal specimens just before and a week after the start of antibiotic treatment (i.e. on the seventh day).

The 16S rRNA gene sequence analysis on fecal samples taken before treatment identified 356 unique microbial taxa out of the 21 samples, with an average of 143 taxa per individual. As expected, gut microbiota was dominated by four bacterial phyla: Firmicutes (65%), Bacteroidetes (28%), Proteobacteria (5%) and Actinobacteria (2%). The number of the detected groups, from phyla to species level, is given in **Table 1**.

**Table 1:** Number of microbial groups at different taxonomic levels.

BF_ATB	Number of microbial groups detected	Most abundant group
Phyla	7	Firmicutes
Class	12	Clostridia
Order	19	Clostridiales
Family	40	Ruminococcaceae
Genus	68	*Bacteroides*
Species	356	*Faecalibacterium prausnitzii*

BF_ATB  =  Before antibiotic treatment.

Twenty-nine bacterial taxa, which were shared by more than 80% of the subjects, constituted the core phylogenetic microbiota and accounted for 44% of the sequences, *Faecalibacterium prausnitzii* (7.2%) being the most abundant (**Table 2**).

**Table 2:** Proportion of sequences for each OTU of the phylogenetic core before and after antibiotics.

Consensus Lineage	Proportion sequences before antibiotics	Proportion of sequences after antibiotics
Bacteroidetes;Bacteroidaceae;Bacteroides #46	2.87	10.74
Bacteroidetes;Bacteroidaceae;Bacteroides #569	1.41	9.86
Bacteroidetes;Bacteroidaceae;Bacteroides uniformis	3.06	4.58
Bacteroidetes;Rikenellaceae #1950	0.81	2.88
Firmicutes;Ruminococcaceae;Faecalibacterium prausnitzii	1.78	2.58
Bacteroidetes;Bacteroidaceae;Bacteroides #1010	ND	1.62
Bacteroidetes;Porphyromonadaceae;Parabacteroides distasonis	0.23	1.33
Firmicutes;Lachnospiraceae;Ruminococcus torques	2.18	1.03
Firmicutes;Lachnospiraceae;Blautia #1159	2.57	0.79
Bacteroidetes;Bacteroidaceae;Bacteroides #1698	ND	0.50
Firmicutes;Lachnospiraceae #1872	0.44	0.42
Firmicutes;Lachnospiraceae;Ruminococcus #789	0.34	0.18
Firmicutes;Ruminococcaceae;Faecalibacterium prausnitzii	5.42	ND
Bacteroidetes;Bacteroidaceae;Bacteroides #529	3.04	ND
Firmicutes;Ruminococcaceae;Oscillospira #1434	2.67	ND
Bacteroidetes;Rikenellaceae #531	2.30	ND
Firmicutes;Lachnospiraceae;Roseburia faecis	2.29	ND
Firmicutes;Lachnospiraceae;Coprococcus #11	1.88	ND
Firmicutes;Ruminococcaceae;Ruminococcus #1267	1.63	ND
Firmicutes;Ruminococcaceae #109	1.34	ND
Bacteroidetes;Bacteroidaceae;Bacteroides eggerthii	1.13	ND
Firmicutes;Lachnospiraceae;Lachnospira #153	1.08	ND
Firmicutes;Lachnospiraceae;Ruminococcus gnavus	1.01	ND
Firmicutes;Ruminococcaceae #1665	0.85	ND
Bacteroidetes;Bacteroidaceae;Bacteroides #1803	0.76	ND
Firmicutes;Ruminococcaceae;Oscillospira #1304	0.74	ND
Firmicutes;Ruminococcaceae #1470	0.73	ND
Firmicutes;Lachnospiraceae;Blautia #1299	0.67	ND
Firmicutes;Lachnospiraceae;Dorea formicigenerans	0.35	ND
Firmicutes;Ruminococcaceae #1866	0.21	ND
Firmicutes;Lachnospiraceae;Blautia #2036	0.20	ND

ND =  Not detected.

#number indicates an arbitrary identification for an OTU.

### Microbiota alteration after treatment

**Figure 1:**
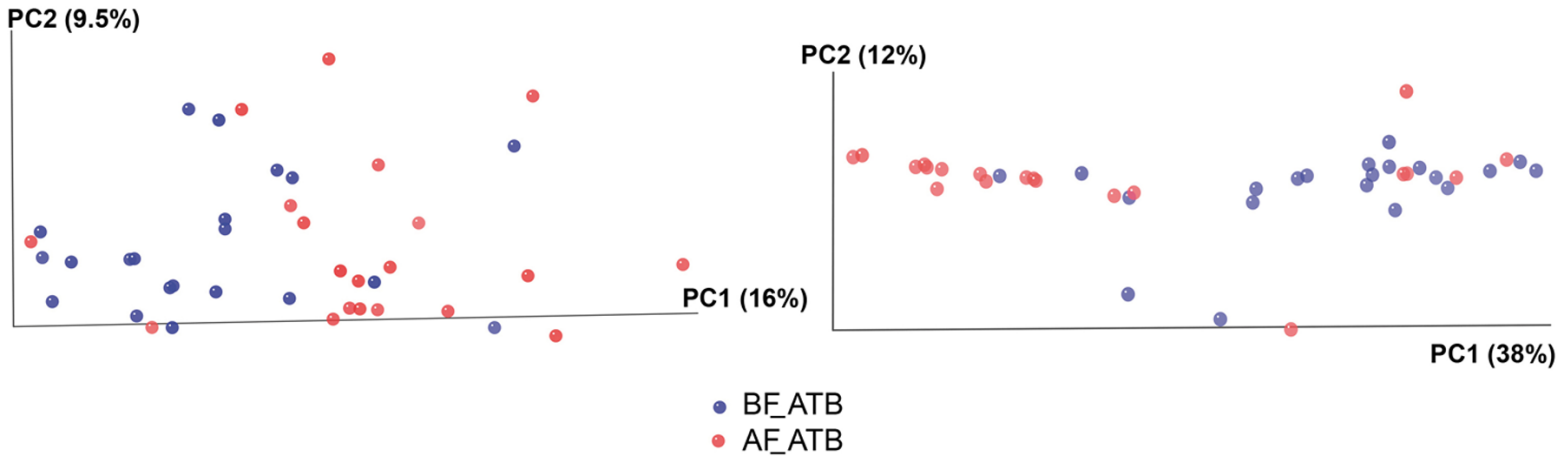
Global effect of antibiotic treatment on fecal microbiota. Communities clustered using PCoA of the unweighted (on the left) and weighted (on the right) UniFrac distance matrix. Only the two first principal components are shown. Each dot represents the whole microbiota of a fecal sample. BF_ATB  =  Before antibiotic treatment and AF_ATB  =  After antibiotic treatment (N = 21).

On the basis of the 16S rRNA sequence analysis and taking into account all the types of antibiotics, we observed that seven days of treatment caused a global change in microbial community structure, as attested by the separate clustering of samples before and after antibiotic intake with both weighted and unweighted UniFrac methods (i.e. taking into account both composition and abundance of the species or only the composition, respectively, **Figure 1** and **Figure S2**). These methods measure similarity between microbial communities on the basis of the degree to which their component taxa share branch length on a bacterial tree of life. This observation indicates that microbial abundance and composition were affected by the antibiotic treatments. However, the clustering was stronger with weighted (38%) than unweighted (15%) UniFrac; implying that antibiotics affected both abundance and composition, and not only the latter.

**Figure 2:**
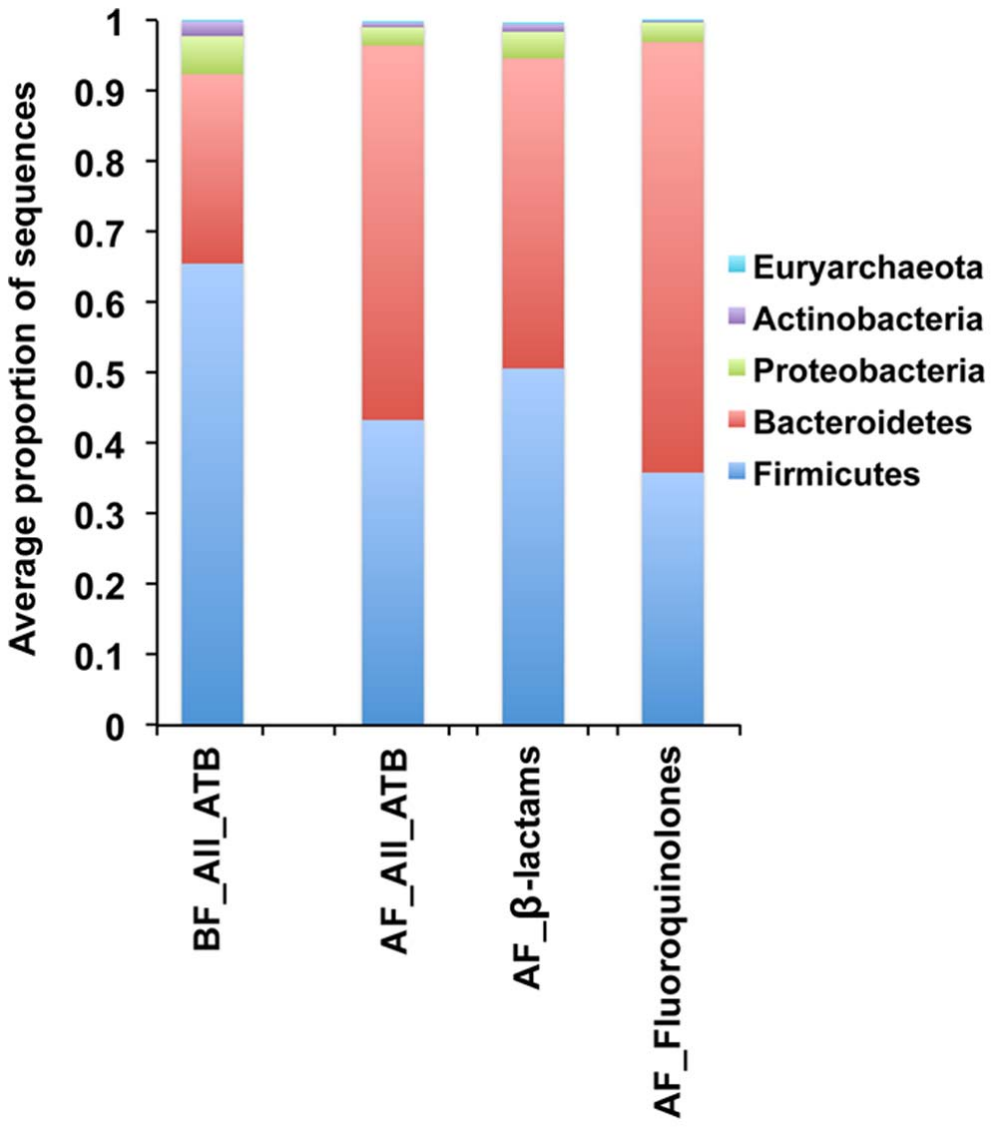
Microbial composition at the phylum level based on 16S rRNA gene sequences. BF  =  Before treatment; AF  =  After treatment; ATB  =  Antibiotics. For all antibiotics N = 21; for β-lactams N = 11; for fluoroquinolones N = 10.

The core phylogenetic microbiota fell from 29 to 12 microbial taxa, these accounting for 36% of the sequences and shifting from *Faecalibacterium* to *Bacteroides* as the most dominant genus. From the 12 microbial taxa constituting the core, two taxa from the *Bacteroides* genus were new compared to the core before treatment (**Table 2**). Indeed, *Bacteroides* genus increased by 2.5-fold (*p* = 0.0003, *FDR* = 0.016). At the phylum level, both types of antibiotics (β-lactams and fluoroquinolones) tested caused a decrease of Firmicutes and increase of Bacteroidetes (*p*<0.001; *FDR* = 0.002) (**Figure 2**). Antibiotics alone or in combination, as depicted in **Figure S3**, also increased the Bacteroidetes/Firmicutes ratio except for two of the drug combinations: piperacilin/tazobactam and levofloxacin/metronidazole. Piperacilin/tazobactam is a combination of two drugs, which inhibits peptidoglycan subunit synthesis (piperacilin) and β-lactamase (tazobactam). Levofloxacin/metronidazole is a combination of two drugs, both inhibiting enzymes involved in nucleic acid synthesis.

**Figure 3:**
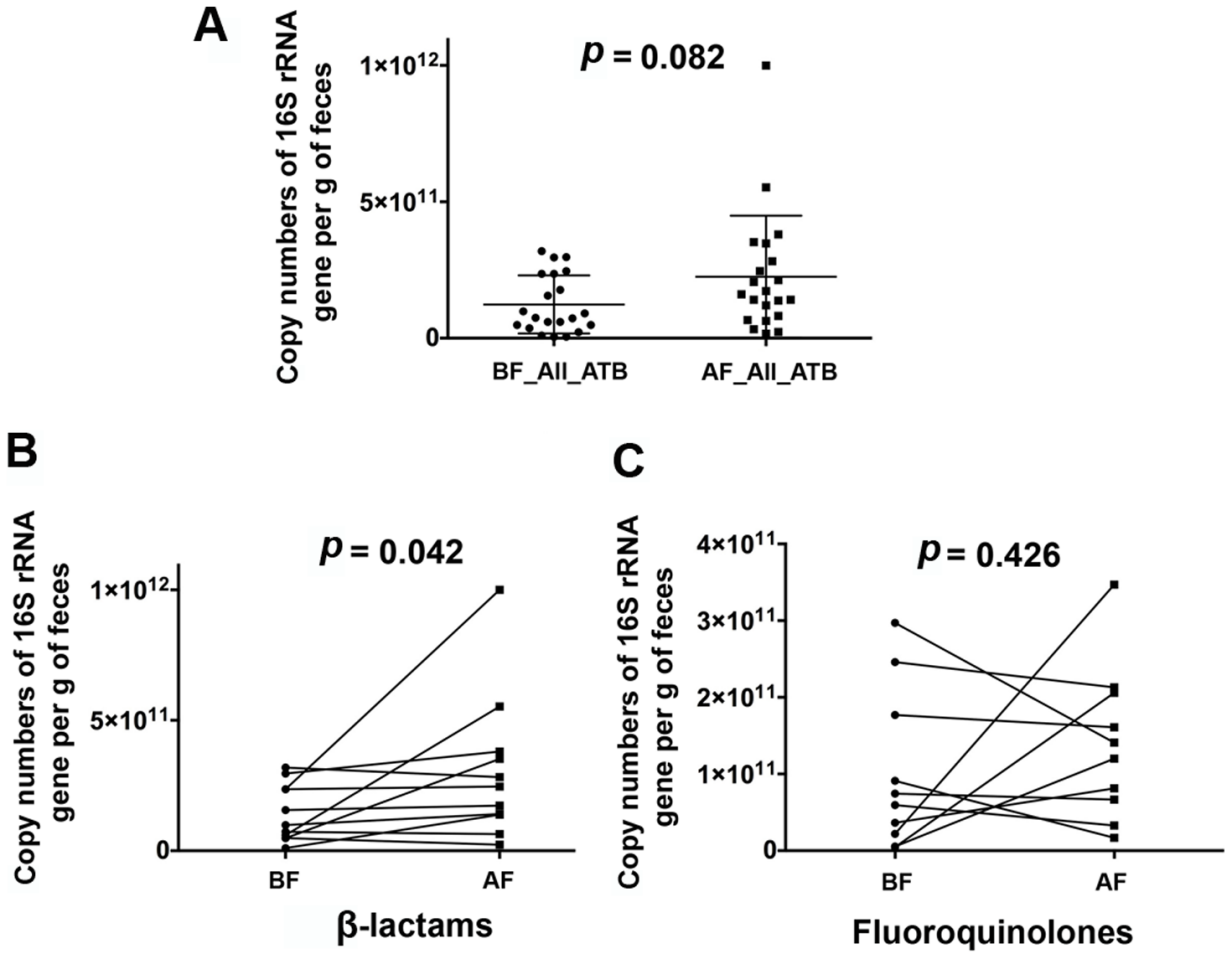
Microbial load as assessed by quantitative real-time PCR (qPCR) of the 16S rRNA gene. (**A**) Comparison of the microbial load between samples before (BF) and after (AF) treatment by both type of antibiotics (All_ATB). Data were compared using Wilcoxon matched-pairs test. (**B**) Comparison of the microbial load between before and after β-lactams treatment. Data were compared using Wilcoxon matched-pairs test. (**C**) Comparison of the microbial load before and after fluoroquinolone treatment. Data were compared using paired t-test. In all tests *p*<0.05 is considered significant. For all antibiotics N = 21; for β-lactams N = 11; for fluoroquinolones N = 10.

**Table 3** shows that antibiotics reduced microbial diversity, as evidenced by the significant decrease in the average number of taxa observed and the Chao1 metric of richness by approximately one fourth. Surprisingly, using quantitative PCR (qPCR) of the 16S rRNA gene, we observed that antibiotic intake did not decrease the microbial load, but instead showed a tendency to increase this parameter, as indicated by the increase in the copy numbers of the 16S rRNA gene (**Figure 3A**, *p* = 0.082; Wilcoxon matched-paired test).

**Table 3:** Microbial richness as assessed by the number of observed taxa and the Chao1 index.

	All subjects (N = 21)			Subjects treated with amoxiclav (N = 7)			Subjects treated with levofloxacin (N = 8)		
	BF_ATB	AF_ATB	*p*	BF_ATB	AF_ATB	*p*	BF_ATB	AF_ATB	*p*
No. of observed taxa	140	105	<0.001	142	117	0.05	137	102	0.03
Chao1 index	179	143	<0.001	188	160	0.04	173	140	0.009

BF_ATB  =  Before antibiotic; AF_ATB  =  After antibiotic. *p* = p values.

### Effect of β-lactams

β-lactam antibiotics interfere with cell wall synthesis by binding to penicillin-binding proteins (PBPs) located in bacterial cell walls. Inhibition of PBPs leads to suppression of peptidoglycan synthesis and finally to cell death [22]. β-lactams show broad-spectrum activity against Gram-negative and Gram-positive bacteria and have been used for a wide range of indications in clinical practice [23]. Sequence analysis revealed that this type of antibiotic significantly increased the proportion of Bacteroidetes 1.5 fold (*p* = 0.019; *FDR* = 0.095; **Figure 2**). No particular taxon from this phylum was significantly affected by the treatment. Quantitative PCR showed that β-lactams doubled the microbial load from 1.4E+11 to 3E+11 16S rRNA copy number per g of faeces (Wilcoxon matched-pairs test, *p = *0.042; **Figure 3B**).

From the 11 patients with β-lactams treatment, 7 of them took amoxicillin-clavulanate (amoxiclav). This drug combines amoxicillin, a β-lactam antibiotic and clavulanic acid, a β-lactamase inhibitor. Clavulanic acid inactivates bacterial β-lactamase and is used to enhance the antibacterial action of β-lactam antibiotics [24]. Sequence analysis showed that amoxiclav, as with β-lactams, significantly decreased microbial diversity metrics (Chao1 and observed species) by around 20% (**Table 3**) while increasing the ratio of Bacteroidetes/Firmicutes. Unlike all β-lactams together, this antibiotic showed an effect at lower taxonomic levels. It increased Bacteroidia (*p* = 0.0005, *FDR* = 0.004) and Bacteroidales groups of Gram-negative bacteria (*p* = 0.0005, *FDR* = 0.005). At the species level, it induced a 20-fold increase in the proportion of an unknown taxon from the *Bacteroides* group. As for β-lactams, qPCR showed that amoxiclav doubled the microbial load from 1.86E+11 to 3.68E+11 16S rRNA copy number per g of faeces (Wilcoxon matched-paired test, *p = *0.07).

### Effect of fluoroquinolones

Fluoroquinolones are broad-spectrum antibacterial agents; however, they show limited activity against anaerobic bacteria [25]. They play a marked role in treatment of nosocomial bacterial infections. They are often used to treat intracellular pathogens such as *Legionella pneumophila* and *Mycoplasma pneumoniae* [26]. They inhibit the bacterial DNA gyrase (Gram-negative) and topoisomerase IV (Gram-positive) [27], [28]. The 10 patients who took fluoroquinolones presented a significant increased ratio of Bacteroidetes (*p*<0.0001; *FDR*<0.001, **Figure 2**). But, unlike β-lactams, fluoroquinolones did not significantly increase the microbial load (**Figure 3C**). It also affected the gut microbiota down to the species level, by increasing 3 unknown taxa from the *Bacteroides* genus (*p*<0.001; *FDR*<0.08).

Out of 10 patients who took fluoroquinolones, 8 of them took levofloxacin, which has activity against Gram-positive and Gram-negative aerobic bacteria and atypical respiratory pathogens [29]. It is used to treat respiratory, urinary tract, gastrointestinal, and abdominal infections [30]. Levofloxacin also increased the Bacteroidetes/Firmicutes ratio and decreased bacterial diversity by 25%. Like all fluoroquinolones tested, levofloxacin did not cause a clear increase of the microbial load. However, it significantly affected 14 bacterial taxa, out of which 10 unknown *Bacteroides* and 1 unknown *Coproccocus* were 3 to 56-fold increased and 1 unknown *Blautia* was 2-fold decreased (*p*<0.01; *FDR*<0.09) (**Table S2**).

## Discussion

Here we used qPCR and 454 pyrosequencing of the 16S rRNA gene to analyze the short-term effect of fluoroquinolone and β-lactam antibiotics on gut microbiota. Our results show that seven days of treatment greatly and globally disturbed the composition and structure of the gut microbial community. Indeed, regardless of the antibiotic type, our results showed that a decrease in the number of microbial taxa by approximately 25% was associated with an increase in Bacteroidetes groups (Gram-negative bacteria). More specifically, although previous works have shown that species from the *Bacteroides* genus such as *B*. *fragilis* were relatively sensitive to both amoxiclav and levofloxacin [24], [31], [32] using culture methods, our study revealed that both drugs significantly increased several taxa from this genus.

Not surprisingly, our results are concordant with previous studies regarding the reduction in gut microbiota diversity. This decrease appears to be a common trait, independent of the type or dosages of antibiotics or the experimental model used (human/animals) [5], [7], [10], [12], [33].

However, our results contradict the general opinion regarding the effect of broad-spectrum antibiotics on gut microbiota. Indeed, instead of causing a decrease in both Gram-positive and Gram-negative bacteria [26], these drugs induce a significant increase in the latter.

Moreover, our unexpected results, which did not show a decrease in microbial load but instead a tendency towards an increase, are discordant with previous studies using either qPCR [10], [12] or culture methods [34], [35], [36], [37] (**Table S1**). These authors reported, as expected, a significant decrease in microbial load after 3 to 7 days antibiotic intake. To the best of our knowledge, studies using qPCR to measure microbial load with respect to antibiotic studies in human adults have not been reported.

The discrepancy between our study in humans in terms of microbial diversity or load and those performed in animal models could be due to the differences in the type of antibiotics and in the relative dosage administered. Indeed, for instance, Antonopoulos et al [33], used around 2500 fold more concentrated antibiotics (amoxicillin/metronidazole/bismuth) in a mouse model than in the present study. Ourselves, in a previous work using a rat model, we used 17 fold more concentrated antibiotics (vancomycin/imipenem) [10].

Our study, although using a very high-throughput technique to study the effect of β-lactams and fluoroquinolones on the human microbiome, presents several limitations. Indeed, considering that this work involved participants with specific criteria of recruitment, we used a relatively small cohort. Furthermore, the design for future studies should also include a questionnaire related to diet or probiotic intake, in order to exclude any external contributing factors. Moreover, by analyzing only two samples per participant, we did not perform a longitudinal study, which were already published by previous research groups [4], [5]. Finally, our study does not distinguish the presence of viable from non-viable bacteria, which could be solved, in the future, by using a PCR-based method using propidium monoazide [38].

The maintenance of or possible increase in microbial load associated to a decrease in diversity suggest that eviction of microorganisms sensitive to these group of antibiotics provides space for resistant strains to overgrow and dominate the niche. This microbial reshaping due to differential sensitivity to antibiotics might explain why resilience is not complete long after treatment cessation. Therefore the systematic use of these antibiotics could reshape the microbiota in favour of resistant bacterial strains in the long-term. Future studies involving other types of antibiotic could help understanding if the effects in this study could be generalized to other antibiotics.

## Supporting Information

Figure S1 Rarefaction curves of OTU richness based on Chao1 estimation in feces samples of patients before (BF_ATB) and after antibiotic treatment (AF_ATB).
(TIF)

Figure S2 Global effect of antibiotic treatment on fecal microbiota. Communities clustered using PCoA of the unweighted (on the left) and weighted (on the right) UniFrac distance matrix. Only the two first principal components are shown. BF_ATB and AF_ATB  =  Before and after antibiotic treatment (N = 21). Each dot represents the microbial community of a sample, and dots representing samples from the same patient were connected by a line.
(TIF)

Figure S3 Microbial composition at the phylum level based on 16S rRNA gene sequences. BF and AF refer to before and after antibiotic treatment and N is the number of subjects.
(TIF)

Table S1 Literature search for studies related to effect of antibiotics on the gut microbiota.
(DOC)

Table S2 Microbial taxa affected by levofloxacin.
(DOC)
