# Supplementary figures and images for: Short-Term Effect of Antibiotics on Human Gut Microbiota
Source: PLoS One. 2014 Apr 18;9(4):e95476. doi: 10.1371/journal.pone.0095476 (PMC3991704; doi:10.1371/journal.pone.0095476)

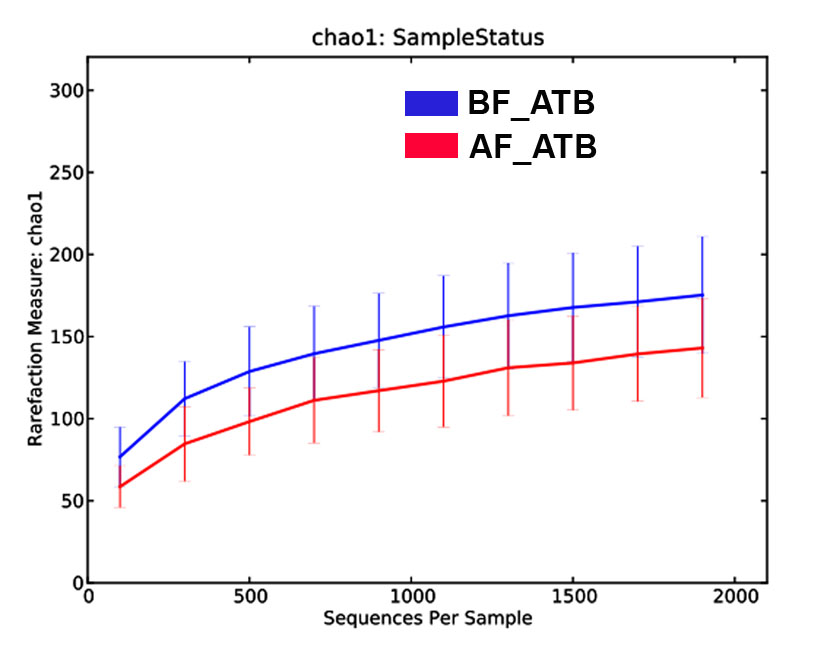

Supplement: Figure S1 — Rarefaction curves of OTU richness based on Chao1 estimation in feces samples of patients before (BF_ATB) and after antibiotic treatment (AF_ATB). (TIF) [file pone.0095476.s001.tif]

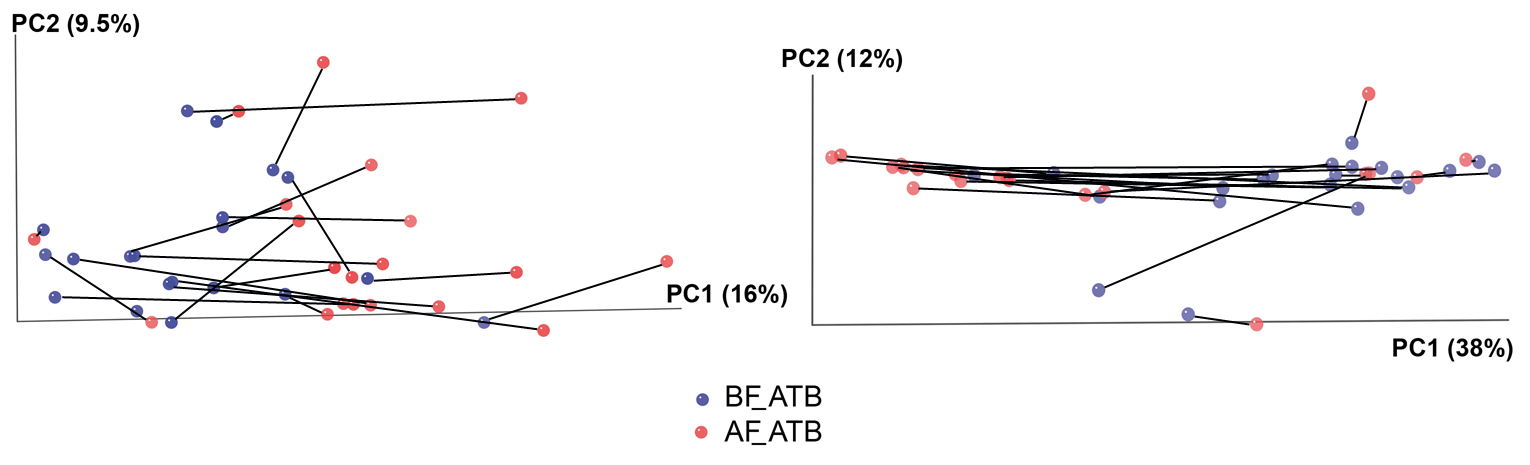

Supplement: Figure S2 — Global effect of antibiotic treatment on fecal microbiota. Communities clustered using PCoA of the unweighted (on the left) and weighted (on the right) UniFrac distance matrix. Only the two first principal components are shown. BF_ATB and AF_ATB = Before and after antibiotic treatment (N = 21). Each dot represents the microbial community of a sample, and dots representing samples from the same patient were connected by a line. (TIF) [file pone.0095476.s002.tif]

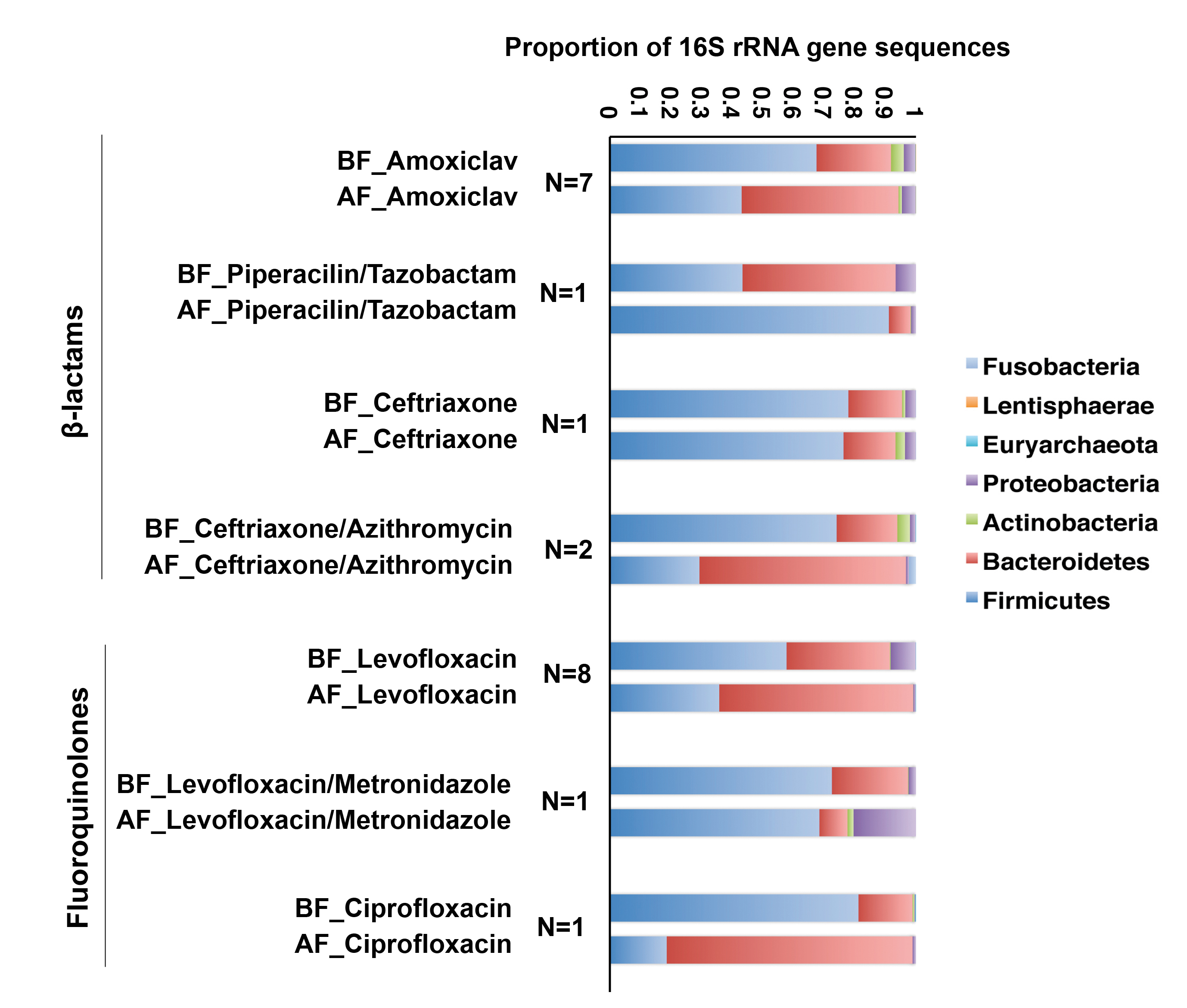

Supplement: Figure S3 — Microbial composition at the phylum level based on 16S rRNA gene sequences. BF and AF refer to before and after antibiotic treatment and N is the number of subjects. (TIF) [file pone.0095476.s003.tif]
