# Supplementary material for: Short-Term Effect of Antibiotics on Human Gut Microbiota
Source: PLoS One. 2014 Apr 18;9(4):e95476. doi: 10.1371/journal.pone.0095476 (PMC3991704; doi:10.1371/journal.pone.0095476)
Supplement: Table S1 — Literature search for studies related to effect of antibiotics on the gut microbiota. (DOC) [file pone.0095476.s004.doc]

**Supporting Table S1. Literature search for studies related to effect of antibiotics on the gut microbiota**

| **Ref** | **Subjects/animal** | **Antibiotic** | **Time points** | **Type of sample** | **Extraction technique** | **Technique** | **Average number of reads** | **Main results of the study** | **Results of microbial load/ count** |
| --- | --- | --- | --- | --- | --- | --- | --- | --- | --- |
| 4 | 3 healthy adults | 500mg ciprofloxacin (fluoroquinolone) twice a day for 5 days | –60, –6, –2, –1 days (pre-treatment), days 3, 5, 33, 180 (post-treatment) | Stool | Bead-beating followed by QIAamp DNA Stool Mini Kit (Qiagen) | Near-complete 16S rDNA and V6 amplified 16S rDNA pyrosequencing | 24,000 per sample | **Decrease** of one third of the taxonomic richness, diversity, and evenness of the community, inter-individual variation; Partial resilience after 4 weeks of treatment cessation | No qPCR, no culture |
| 5 | 3 controls / 3 treated adults patients | 400mg metronidazole (nitromidazole), 250mg clarithromycin (macrolide), and 20mg omeprazole (proton pump inhibitor) | 8, 10, 12, 13 days (depending on patient), 1 year, 4 years after treatment | Throat swabs and stool | Throat: DNeasy Tissue Kit (Qiagen)  Stool: Bead-beating followed by FastDNA SPIN Kit for Soil (BIO 101) | V6 amplified 16S rDNA T-RFLP and pyrosequencing | 5000-11,000 per subject | Significant **decrease** in Actinobacteria in both throat and feces immediately after treatment;  Microbiota remained perturbed in some cases for up to four years post treatment | No qPCR, no culture |
| 6 | 9 controls/ 9 treated infants (within 48 h of birth) | Ampicillin (β-lactam) and gentamicin (aminoglycoside) [no data on dosage] | 4 and 8 weeks after treatment | Stool | QIAamp DNA stool mini kit (Qiagen) | V4 amplified 16S rDNA pyrosequencing and qPCR | No data provided | **Increase** of Proteobacteria at week 8 after cessation of treatment | No culture, using qPCR: No significant differences between antibiotic-treated infant samples compared to those for controls at week 4 or week 8; Significant **increase** at week 8, in total 16S rRNA values in the antibiotic-associated samples |
| 7 | 1 patient | Single dose of ampicillin/sulbactam (β-lactam) and 14 days of cefazolin (β-lactam) treatment | 0, 36, 11, 14 days after initiation of treatment and day 40 after cessation of treatment | Stool | QIAamp DNA Stool kit (Qiagen) | Total (16S rDNA) and active (16S rRNA) microbiota by V1-V3 amplified pyrosequencing, metagenome, metatranscriptome, metabolome and metaproteome | No data provided | **Decrease** of Gram-negative bacteria at day 6;  Increase of active *Parabacteroides* at day 14; minimum richness at day 11; major metabolic changes at day 6 | No qPCR, no culture |
| 8 | 4 healthy controls/ 4 healthy received antibiotics | 150mg clindamycin (lincosamide) 4 times a day for 7 days | 0, 7, 14 days, 3, 6, 9, 12, 18, 24 months after treatment | Stool | FastDNA  SPIN Kit for Soil (Q-BIOgene) | T-RFLP, RT-PCR | No data provided | *Bacteroides* showed sharp **decrease**; Samples at day 0 clearly separated from the rest of the samples; Microbiota stabilizes to pre-clindamycin administration levels after three months post exposure | No culture, no qPCR for bacteria, but RT-PCR of *erm* genes showed **increase** at later time-points compared to basal |
| 9 | 6 healthy | 500mg amoxicillin (β-lactam) per day for 5 days | 0,1, 30, 60 days for all after treatment, day 2, 3, 4 for some | Stool | Godon method [see reference 9] | V6-V8 amplified 16S rRNA gene TTGE, sequencing by Sanger method of specific gel bands | No data provided | Dominant species markedly **decreased** within 2 to 3 days; These tended to return to initial profile within 60 days; Modifications persisted for at least 2 months | No qPCR, no culture |
| 10 | 3 control rats/ 3 rats with antibiotics | 50mg/kg/day vancomycin (glycopeptide) and 50mg/kg/day imipenem (β-lactam) for 3 days | -3 days (pre-treatment), 3 days, 1, 3 months post- treatment | Stool | Bead-beating followed by modified QIAamp DNA stool mini kit (Qiagen) | V4 amplified 16S rRNA gene Pyrosequencing, qPCR | 546,230 reads total, 2000 reads per sample | **Decreased** bacterial phylotype richness controls clustered separately than treated; Major reduction of Bacteroides and Firmicutes; One month later, fecal samples regained similar bacterial load to the controls; Bacteroidetes and Firmicutes recovered as the two major phyla; Bacterial diversity was not resilient | No culture, using qPCR: 10-fold **decrease** in bacterial load of antibiotic treated rats compared with control rats |
| 35 | 8 mice | 1g/ml ampicillin (β-lactam), 0.5g/ml vancomycin (glycopeptide), 1g/ml neomycin sulfate (aminoglycoside), and 1g/ml metronidazole (nitromidazole) | No data | Intestines, Stool | Culture in aerobic/ anaerobic conditions | CFU counts | Not applicable | Oral treatment with antibiotics significantly **decreased** the bacterial counts present in fecal and intestinal samples; In aerobic and anaerobic conditions a significant **decrease** of bacterial counts was found after 1 wk of treatment; antimicrobial treatment did not completely deplete bacterial presence, showing that certain bacterial populations remain viable despite antibiotic treatment | No qPCR, **Decrease** in the bacterial counts of fecal and intestinal samples by culture |
| 36 | Mice | 0.5mg/ml cefoperazone (β-lactam) for 5 days | Day 5 | Stool | Culture | CFU counts | Not applicable | Culturable anaerobic and enteric bacteria levels in the gut was **decreased** by 99.99% at day 4 of antibiotic treatment; When antibiotic treatment was discontinued, the numbers of both anaerobic and enteric bacteria increased | No qPCR, **decrease** in the bacterial counts at day 4 by culture |
| 37 | Mice | 1g/ml ampicillin (β-lactam), 0.5g/ml vancomycin (glycopeptide), 1g/ml neomycin sulfate (aminoglycoside), and 1g/ml metronidazole (nitromidazole) for 7 days | Day 7 | Intestinal and stool samples | Culture | CFU counts | Not applicable | Oral antibiotic treatment significantly **decreased** the total bacterial numbers recovered | No qPCR, **decreased** bacterial colonies |
