# Supplementary material for: Short-Term Effect of Antibiotics on Human Gut Microbiota
Source: PLoS One. 2014 Apr 18;9(4):e95476. doi: 10.1371/journal.pone.0095476 (PMC3991704; doi:10.1371/journal.pone.0095476)
Supplement: Table S2 — Microbial taxa affected by levofloxacin. (DOC) [file pone.0095476.s005.doc]

**SUPPORTING INFORMATION**

**Supporting Table S2**. **Microbial taxa affected by levofloxacin**

| **FDR corrected** | **Proportion of sequences** | | **Ratio** | **Taxa** |
| --- | --- | --- | --- | --- |
|  | Before levofloxacin | After levofloxacin | **after:before** Levofloxacin |  |
|  |  |  |  |  |
| 0.0024 | 0.0001 | 0.0033 | 30.42 | Unknown *Bacteroides* |
| 0.0253 | 3.2e-05 | 0.0018 | 56.72 | Unknown *Bacteroides* |
| 0.0206 | 0.0001 | 0.0049 | 34.79 | Unknown *Bacteroides* |
| 0.0364 | 0.0002 | 0.0048 | 28.72 | *Bacteroides plebeius* |
| 0.0322 | 6.4e-05 | 0.0017 | 27.50 | Unknown *Bacteroides* |
| 0.0329 | 0.0144 | 0.1651 | 11.49 | Unknown *Bacteroides* |
| 0.0386 | 0.005 | 0.0785 | 15.79 | Unknown Bacteroideceae |
| 0.0491 | 0.0002 | 0.001 | 5.66 | Unknown *Coprococcus* |
| 0.0507 | 0.0072 | 0.0855 | 11.82 | Unknown Bacteroideceae |
| 0.0582 | 0.0018 | 0.0097 | 5.32 | Unknown *Bacteroides* |
| 0.0582 | 0.0004 | 0.0015 | 3.39 | Unknown *Bacteroides* |
| 0.0695 | 0.0008 | 0.0067 | 8.49 | Unknown *Bacteroides* |
| 0.0654 | 0.0021 | 0.0001 | 0.05 | Unknown *Blautia* |
| 0.0899 | 0.0004 | 0.0016 | 4.08 | Unknown Ruminococcaceae |
| 0.0886 | 0.0015 | 0.0096 | 6.57 | Unknown *Bacteroides* |
